# Efficacy and immunological changes of sublingual immunotherapy in pediatric allergic rhinitis

**DOI:** 10.1016/j.waojou.2023.100803

**Published:** 2023-07-23

**Authors:** Yinhui Zeng, Haiqing Xiao, Shengli Gao, Jinyuan Li, Chao Yang, Qingxiang Zeng, Xi Luo, Renzhong Luo, Xi Chen, Wenlong Liu

**Affiliations:** a Department of Otolaryngology, Guangzhou Women and Children's Medical Center, Guangzhou Medical University, Guangdong Provincial Clinical Research Center for Child Health, Guangzhou, 510623, China; b Department of Anesthesiology, Guangzhou Women and Children's Medical Center, Guangzhou Medical University, Guangdong Provincial Clinical Research Center for Child Health, Guangzhou, 510623, China

**Keywords:** Rhinitis, Allergic, Cytokines, Efficacy, Sublingual immunotherapy, Treatment outcome

## Abstract

**Background:**

Allergen-specific immunotherapy, including subcutaneous immunotherapy (SCIT) and sublingual immunotherapy (SLIT), improves the disease progression of allergic rhinitis (AR). SCIT and SLIT exhibit similar efficacy, but SLIT has less systemic reactions. However, few studies have investigated the underlying mechanisms of SLIT treatment. In this study, we explored the efficacy of SLIT under different treatment durations and immunological changes.

**Methods:**

This retrospective study was conducted from August 2017 to August 2022 in our hospital. A total of 314 children who underwent SLIT were divided into the following groups based on their treatment duration: the 1 year group (6 months–1 year), the 2 years group (1–2 years), and the 3 years group (2–3 years). The treatment efficacy was confirmed using a combined symptom and medication score (SMS). Multiple serum cytokines were measured using Luminex. Various immune cells in PBMCs were determined using flow cytometry.

**Results:**

The total nasal symptom score (TNSS), rescue medication score (RMS), and SMS of the 3 years group was significantly different from those of the 1 years and 2 years groups. At the end of the 2 years following cessation of SLIT, the following results were observed in the 3 years group: 1) the TNSS, RMS, and SMS had significantly improved, 2) the serum IL-10, TGF-beta, and IL-35 levels had increased significantly, and 3) the percentages of regulatory T cell, regulatory B cell, and follicular regulatory T cell increased significantly.

**Conclusion:**

Our results suggest that 3 years of SLIT is necessary for long-term effects and continued immunological changes.

## Introduction

The incidence of allergic rhinitis (AR) has significantly increased in China over the last few decades.^1^^,^^2^ A recent study reported that the occurrence of AR ranged from 9.6% to 23.9% in 18 major cities in China.^1^ Allergen avoidance, medications aimed to relieve symptoms, allergen-specific immunotherapy (AIT), and health education are 4 methods of comprehensive treatment.^3^

AIT, including subcutaneous immunotherapy (SCIT) and sublingual immunotherapy (SLIT), is currently the only treatment to improve the disease progression of AR.^4^ Compared with SCIT, SLIT has similar efficacy and less systemic reactions.^5^^,^^6^ Several studies have proven that 3 years of AIT induces long-lasting effects, even after cessation.^7^, ^8^, ^9^ The mechanisms of successful AIT include reduced allergen specific T- and B-cell inflammation, decreased IgE production, and inhibited mast cell and basophil activation, while the IgG4 production, the function of T regulatory cells and B regulatory cells were enhanced.^10^ However, few studies have investigated the underlying mechanisms of treatment with SLIT in children.

In this study, we aimed to explore the efficacy of SLIT under different treatment durations and immunological changes.

## Methods

### Patients

We conducted a retrospective study from August 2017 to August 2022 in our hospital. The AR was determined according to the Allergic Rhinitis and its Impact on Asthma (ARIA) guidelines.^11^ This included typical allergic symptoms such as runny nose, itchy nose, sneezing, nasal block, positive skin-prick test (SPT), and/or serum-specific immunoglobulin E (IgE) for *Dermatophagoïdes farina* or accompanied by *Dermatophagoïdes pteronyssinus*. All recruited subjects were children (4–18 years old) and the disease duration was longer than 1 year. Children with asthma, nasal polyposis, immunodeficiency, severe systematic diseases, or sensitivity to allergens except for *Dermatophagoïdes farina* or *Dermatophagoïdes pteronyssinus* were excluded. Asthma was excluded using questionnaires on the history of recurrent dyspnea, wheezing, or cough episodes, and methacholine PC20 ≤ 4 mg/mL or albuterol reversibility of FEV1 ≥ 10%. Informed consent was obtained from the children and their parents. Our study obtained approval from local ethics committee boards.

### Immunotherapy and grouping

For SLIT, *Dermatophagoides farinae* drops (Zhejiang Wolwo, China) were provided. The drugs were administered with increasing concentrations from 1 μg/mL to 333 μg/mL over the course of 3 weeks according to the recommendations (1,2, 3, 4, 6, 8, 10 drops of No. 1 to No. 3 drops every week for 3 weeks). In the maintenance stage from the fourth week, 333 μg/mL of drugs (3 drops) were given until the end of the treatment. All of the children's guardians were provided with a detailed and comprehensive health education and were informed the importance of enough treatment period (3 years) and regular follow-up. After 3 years' follow-up, the children were divided into 3 groups according to actual completed treatment duration: 1 year group (6 months–1 year), 2 years group (1–2 years), and 3 years group (2–3 years).

### Efficacy of SLIT

The treatment efficacy and the adverse events of SLIT were assessed using symptom and medication scores at the baseline, at the end of SLIT and at the end of the 2 years following cessation of SLIT using Diary Card throughout the study (at least 3 times every week).^12^ In brief, the rhinorrhea, sneezing, nasal obstruction, and itchy nose were scored from 0 to 3 according to disease severity (0, no symptom; 1, slight symptoms; 2, moderate symptoms; 3, severe symptoms) and the total nasal symptom score (TNSS) was obtained. The rescue medication score (RMS) was defined as 1 point for use of oral or topical antihistamines and 2 points for topical corticosteroids. The combined symptom and medication score (SMS) was used for evaluation of efficacy. The children obtained a 30% SMS decrease compared to their baseline score were defined as response group.

### Collection of serum samples and measurement of Ig and cytokines in serum

Venous blood samples were collected after centrifugation at 3000 *g* for 20 min. The serum was stored at −80 °C for further analysis. Multiple serum cytokines were tested by Luminex. The Ig levels were measured by enzyme linked immunosorbent assay (ELISA) kits (R&D systems, USA) according to the instructions.

### Flow cytometry analyses

Isolation of the peripheral blood mononuclear cells (PBMCs) was performed by density-gradient centrifugation. Various immune cells in PBMCs were determined using flow cytometry. The antibodies used for staining are listed in Table 1.

**Table 1:** Antibodies used in staining of immune cells.

Cells	Antibodies	Company
Th2	IL-4,CD3,CD4,	BD Biosciences
Th17	IL-17,CD3,CD4	BD Biosciences
Treg	CD25,Foxp3,CD4, CD3	BD Biosciences
Breg	IL-10,CD19	BD Biosciences
ILC2	CD127,CRTH2,Lin,FcεRI	eBioscience
Tfh2	IL-4,CXCR5,CD4	BD Biosciences
Tfr	CD3,CD4,CD45RA,CXCR5,CD25,CD127	BD Biosciences

Th2, T helper 2 cell; Th17, T helper 17 cell; Treg, regulatory T cell; Breg, regulatory B cell; ILC2, group 2 innate lymphoid cells; Tfh2, follicular T helper 2 cell; Tfr, follicular regulatory T cell

### PBMC stimulation

The isolated PBMCs (2∗10^6^ cells) cultured in Roswell Park Memorial Institute 1640 medium (Thermo Fisher Scientific) containing 10% fetal bovine serum, 100 U/ml of penicillin, and 100 μg/mL of streptomycin (Thermo Fisher Scientific) were incubated with 10 μg/mL recombinant Der p 1 (Indoor Biotechnologies Ltd) at 37 °C for 5 days. The supernatants were collected for cytokine measurement by Luminex. The cell viability was 98%–100% as determined by Trypan blue exclusion assay. After washing with 2% FBS-containing PBS, cells were stained with fluorescence-labeled Abs (Table 1) for 30 min, washed with PBS, and analyzed by the FACSAria II flow cytometry device (BD Biosciences).

### Statistical analysis

Statistical analysis was performed using GraphPad Prism 9.0 software. A preliminary analysis of the data was performed using normality, homogeneity of variances, firstly. Comparisons between groups were performed by unpaired, non-parametric testing (Kruskal-Wallis test) and chi-square tests. The Intra-Group comparison was performed using a paired *t*-test. P < 0.05 was defined as statistically significant.

## Results

### Baseline characteristics

A total of 314 children were enrolled in this study and 296 children finished the study. The baseline characteristics of subjects are summarized in Table 2. The age, sex ratio, BMI, and disease duration and severity among different groups had no significant difference (*P* > 0.05).

**Table 2:** Baseline characteristics of study subjects.

Characteristics	1 year group	2 years group	3 years group
Cases	107	74	134
Age (years)	7.5 ± 3.5	7.2 ± 2.7	6.7 ± 2.3
Male/female	76/31	52/21	91/43
BMI	16.2 ± 3.6	15.1 ± 3.3	15.3 ± 3.1
Duration of symptoms, (years)	1.8 ± 1.3	1.7 ± 1.5	1.9 ± 1.3
Disease severity (moderate/severe)	43/64	20/54	54/80
Serum sIgE level to Der p (IU/mL)	26.6 (2.1–98.5)	21.3 (9.8–87.3)	19.5 (3.8–95.1)
Serum sIgE level to Der f (IU/mL)	38.2 (3.1–93.8)	31.8 (1.9–66.4)	25.4 (4.9–91.7)
Total IgE (IU/mL)	265.4 (42.1–838.4)	211.8 (25.6–731.4)	315.6 (12.9–679.2)

BMI, body mass index; Der p, Dermatophagoïdes pteronyssinus; Der f, Dermatophagoïdes farina

For the 1 year group of 107 children, 101 of them responded to our survey at 2 years after the cessation of treatment. For the 2 years group of 74 children, 70 of them responded to our survey at 2 years after the cessation of treatment. Lastly, for the 3 years group of 134 children, 125 of them responded to our survey at 2 years after the cessation of treatment. No child developed asthma during the study period. The reasons for not being able to persist in treatment in 1 year and 2 years group were summarized in Table S1.

### Comparison of efficacy among groups

The TNSS, RMS, and SMS at the end of SLIT improved significantly compared with the baseline levels in all groups (Table 3). At the end of SLIT, the TNSS, RMS, and SMS of the 1 year group was significantly higher than the 2 years and 3 years groups, but there was no difference between the 2 years and 3 years groups (Table 3). At the end of the 2 years following cessation of SLIT, the TNSS, RMS, and SMS of the 3 years group had significant improved compared to the 1 year and 2 years groups. The adverse events were summarized in Table 3 and no severe adverse events were reported.

**Table 3:** Clinical efficacy and AEs of SLIT.

	1 year group	2 years group	3 years group
**Baseline score**			
TNSS	6.02 ± 2.56	5.93 ± 2.80	5.65 ± 2.28
RMS	0.92 ± 0.87	0.85 ± 0.88	1.01 ± 0.88
SMS	6.95 ± 2.82	6.78 ± 2.96	6.66 ± 2.47
**The end of SLIT**			
TNSS	3.01 ± 2.04∗	2.39 ± 2.22∗^#^	1.93 ± 1.65∗^#^
RMS	0.21 ± 0.41∗	0.16 ± 0.37∗^#^	0.06 ± 0.25∗^#^
SMS	3.21 ± 2.12∗	2.55 ± 2.38∗^#^	2.01 ± 1.75∗^#^
**2 years after cessation of SLIT**			
TNSS	5.26 ± 2.18^$^	4.21 ± 1.95∗^#$^	1.88 ± 1.72∗^#&^
RMS	0.87 ± 0.76^$^	0.57 ± 0.32∗^#$^	0.05 ± 0.18∗^#&^
SMS	6.48 ± 2.95^$^	4.69 ± 2.21∗^#$^	2.19 ± 1.58∗^#&^
**AEs**			
Allergic symptom	5	3	6
Local irritation symptom	3	3	4
Gastrointestinal symptom	2	1	0
Other minor symptom	2	1	1

SLIT, sublingual immunotherapy; TNSS, total nasal symptom score; RMS, Rescue medication score; SMS, symptom medication score; AEs, adverse events. ∗ Compared with baseline score, *P* < 0.05. # Compared with 1 year group, *P* < 0.05. $ Compared with the end of SLIT, *P* < 0.05. & Compared with 2 years group, *P* < 0.05

### Comparison of Ig and cytokines among groups

**Fig. 1:**
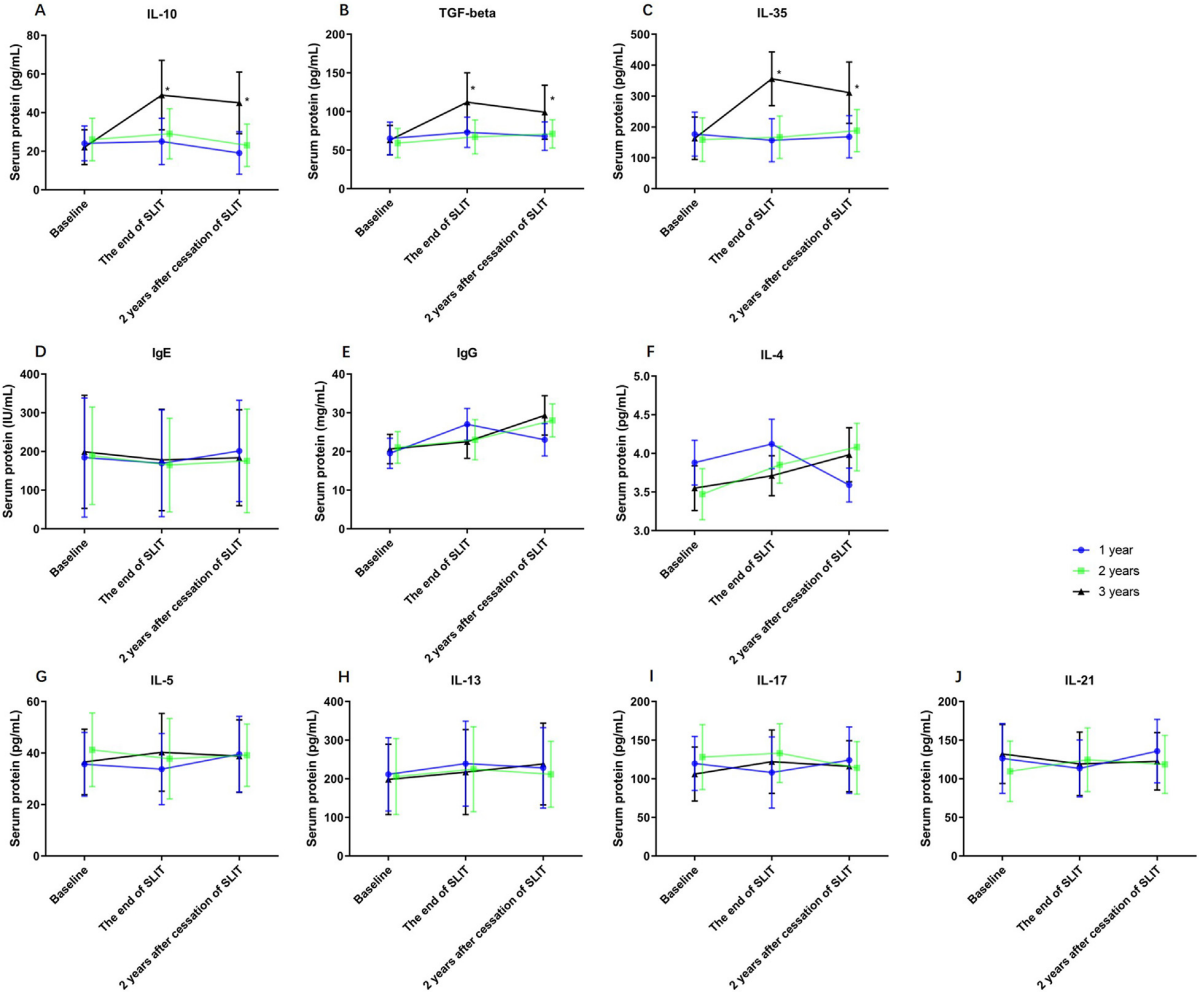
Comparison of serum cytokine levels at the baseline, the end of SLIT and 2 years after the cessation of SLIT. A, IL-10 protein expression. B, TGF-β expression. C, IL-35 expression. D, IgE expression. E, IgG expression. F, IL-4 expression. G, IL-5 expression. H, IL-13 expression. I, IL-17 expression. ∗P < 0.05. Data are expressed as mean ± SEM. SLIT, sublingual immunotherapy.

The serum IL-10, TGF-beta, and IL-35 levels at the end of SLIT and the end of the 2 years following cessation of SLIT in the 3 years group increased significantly compared to the 1 year and 2 years groups (Fig. 1). At the end of the 2 years following cessation of SLIT, the serum IL-10, TGF-beta, and IL-35 levels in the 3 years group were not significantly changed (Fig. 1). The sIgE, IgG, IL-4, IL-5, IL-13, IL-17, and IL-21 levels were not significantly different between groups (Fig. 1). Despite the IL-4 expression decreased at the end of 2 years following cessation, no significant difference was found.

### Comparison of percentage of immune cells among groups

**Fig. 2:**
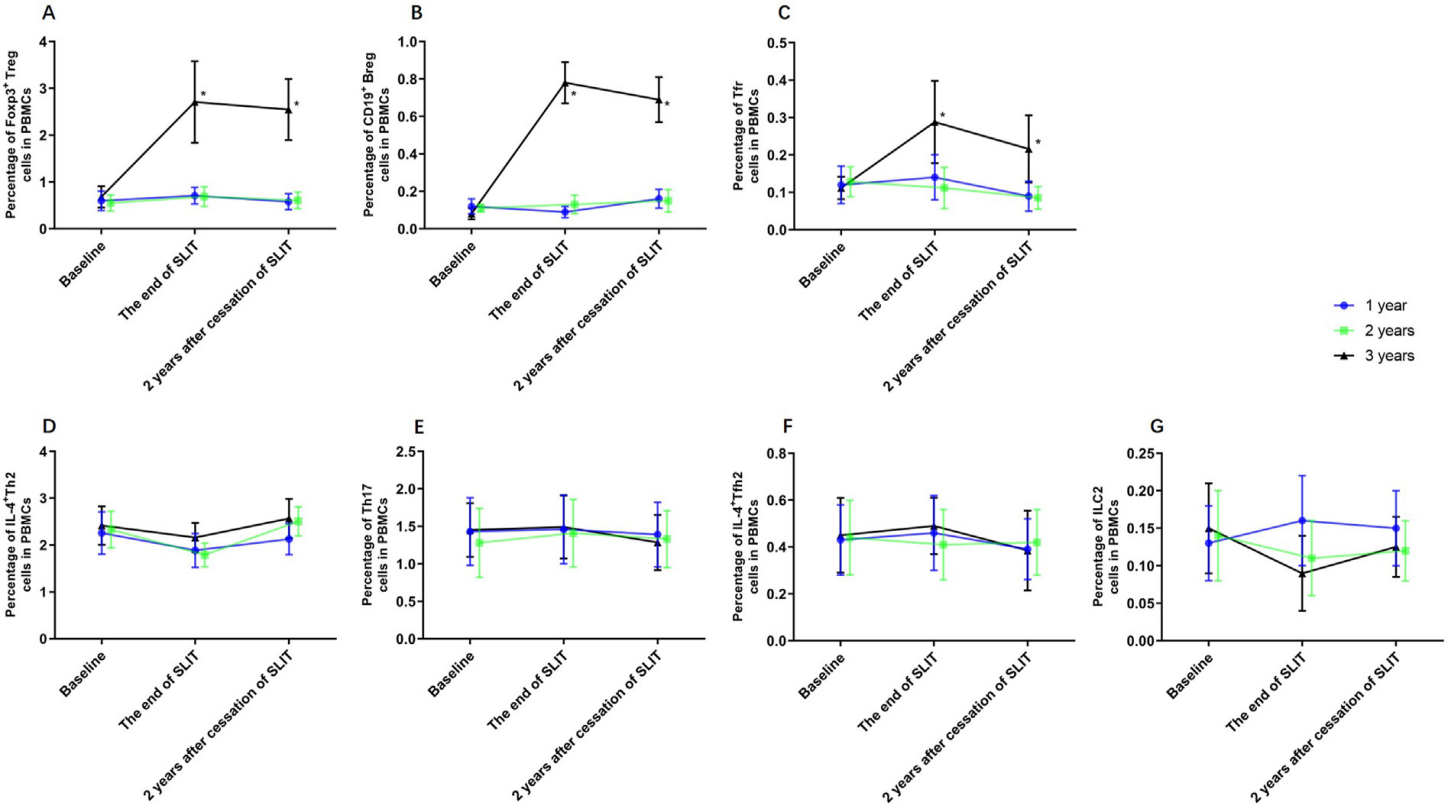
Comparison of percentage of immune cells at the baseline, the end of SLIT and 2 years after the cessation of SLIT. A, Percentage of Foxp3^+^ Treg cells in PBMCs. B, Percentage of CD19^+^ Breg cells in PBMCs. C, Percentage of Tfr cells in PBMCs. D, Percentage of IL-4^+^Th2 cells in PBMCs. E, Percentage of Th17 cells in PBMCs. F, Percentage of IL-4^+^Tfh2 cells in PBMCs. G, Percentage of ILC2 cells in PBMCs. ∗*P* < 0.05. Data are expressed as mean ± SEM. SLIT, sublingual immunotherapy.

**Fig. 3:**
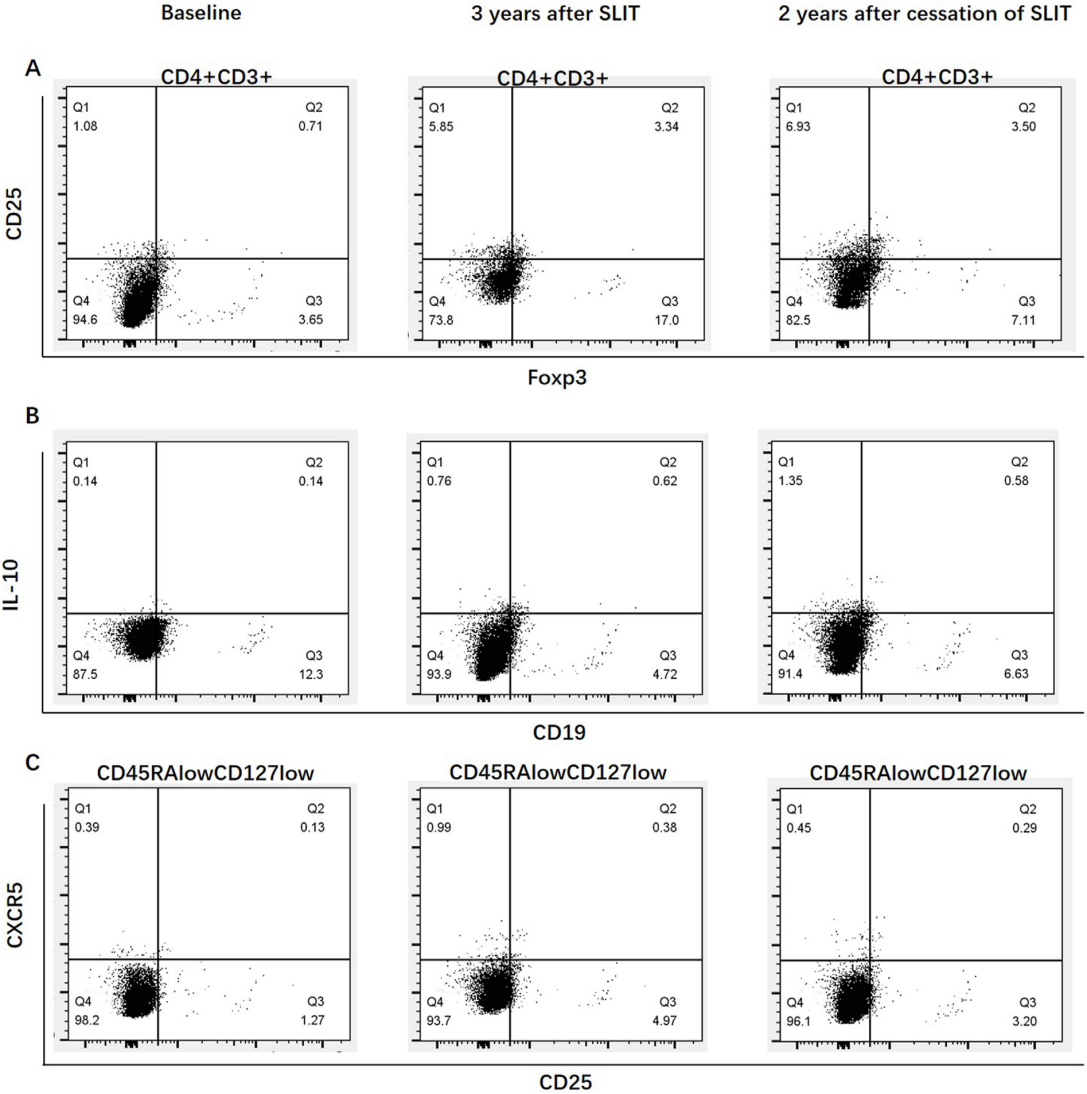
Comparison of Treg, Breg and Tfr at the baseline, the end of SLIT and 2 years after the cessation of SLIT. A, CD4^+^CD3^+^CD25^+^FOXP3^+^ Treg cells. B, CD19^+^IL-10^+^ Bregs cells. C, CXCR5^+^CD25^+^CD45RA^low^CD127^low^ Tfr cells. SLIT, sublingual immunotherapy.

The percentages of regulatory T cell (Treg), regulatory B cell (Breg), and follicular regulatory T cell (Tfr) at the end of SLIT and the end of the 2 years following cessation of SLIT in the 3 years group increased significantly compared to the 1 year and 2 years groups (Fig. 2, Fig. 3). At the end of the 2 years following cessation of SLIT, the percentage of Treg, Breg, and Tfr in the 1 year, 2 years and 3 years group were not significantly changed compared to the end of SLIT (Fig. 2, Fig. 3). The percentages of T helper 2 cell (Th2), T helper 17 cell (Th17), follicular T helper 2 cell (Tfh2), and group 2 innate lymphoid cells (ILC2) at different detection time points of the 1 year, 2 years and 3 years group had no significant differences (Fig. 2). In the 3-year group, ILC2 showed a downward trend at the end of treatment and then moderated but remained lower 2 years after cessation, but no significant difference was found.

### Comparison of cytokines and percentage of immune cells between good responsive and poor responsive groups

To analyze the coorelation between long-term efficacy of SLIT and immunological changes, we compared the immunological parameters between the good responsive and poor responsive groups at the end of the 2 years following cessation of SLIT. Our data showed that the serum IL-10, TGF-beta, IL-35 levels and the percentages of Treg, Breg, Tfr were significantly lower in poor responsive groups compared with good responsive group (Table 4), while other parameters showed no significant difference between groups.

**Table 4:** Immunological parameters between the good responsive and poor responsive groups at the end of the 2 years following cessation of SLIT.

Parameters	Good responsive group	Poor responsive group
IL-4	3.7 ± 1.5	3.1 ± 1.6
IL-5	38.6 ± 21.2	40.3 ± 18.3
IL-13	216 ± 98	178 ± 112
IL-17	108 ± 56	123 ± 64
IL-21	133 ± 87	127 ± 69
IL-10	46.8 ± 17.6	22.6 ± 15.4∗
TGF-beta	97.6 ± 43.8	53.1 ± 22.7∗
IL-35	316 ± 112	178 ± 103∗
Th2%	2.4 ± 0.9	2.6 ± 0.8
Th17%	0.8 ± 0.4	0.7 ± 0.6
Tfh2%	0.4 ± 0.2	0.3 ± 0.1
ILC2%	0.15 ± 0.04	0.13 ± 0.05
Treg%	2.3 ± 1.5	0.5 ± 0.2∗
Breg%	0.7 ± 0.2	0.1 ± 0.05∗
Tfr%	0.2 ± 0.1	0.08 ± 0.02∗

All the unit of cytokines were pg/ml; SLIT, sublingual immunotherapy, Treg, regulatory T cell, Breg, regulatory B cell, Tfr, follicular regulatory T cell, Th2, T helper 2 cell, Th17, T helper 17 cell, Tfh2, follicular T helper 2 cell, ILC2, group 2 innate lymphoid cells. The calculation of Th2, Th17, Tfh and Tfr cells percentage is based on the numbers of CD4^+^ T cell as the denominator. The calculation of Treg, Breg and ILC2 is based on the numbers PBMC as the denominator. ∗ Compared with Good responsive group, *P* < 0.05

### Allergen-induced immunological responses

After stimulated by Recombinant Der p 1, the serum IL-10, TGF-beta, IL-35 levels and the percentages of Treg, Breg, Tfr in the 3 years group were significantly higher compared with baseline levels (Table 5), while other cytokine levels and the percentages of other cells showed no significant difference between groups (Data not shown).

**Table 5:** Comparison of allergen-induced immunological responses between baseline and at the end of SLIT.

Parameters	1 year group	2 years group	3 years group
IL-10	21.6 ± 12.8 *vs* 23.7 ± 13.2	24.5 ± 13.9 *vs* 29.8 ± 11.6	23.7 ± 11.6 *vs* 78.9 ± 22.5∗
TGF-beta	62.3 ± 27.1 *vs* 75.8 ± 31.4	69.4 ± 25.8 *vs* 87.6 ± 28.5	60.6 ± 23.4 *vs* 124.7 ± 53.5∗
IL-35	124 ± 78 *vs* 119 ± 65	133 ± 59 *vs* 156 ± 71	129 ± 63 *vs* 317 ± 122∗
Treg percentage	0.4 ± 0.1 *vs* 0.3 ± 0.2	0.5 ± 0.2 *vs* 0.4 ± 0.1	0.38 ± 0.2 *vs* 2.1 ± 0.6∗
Breg percentage	0.1 ± 0.02 *vs* 0.15 ± 0.05	0.13 ± 0.03 *vs* 0.12 ± 0.03	0.14 ± 0.05 *vs* 0.64 ± 0.08∗
Tfr percentage	0.04 ± 0.01 *vs* 0.03 ± 0.01	0.03 ± 0.02 *vs* 0.05 ± 0.01	0.04 ± 0.02 *vs* 0.21 ± 0.06

All the unit of cytokines were pg/ml; SLIT, sublingual immunotherapy, Treg, regulatory T cell, Breg, regulatory B cell, Tfr, follicular regulatory T cell. ∗ Compared with baseline level, *P* < 0.05

## Discussion

Stable clinical benefits from SLIT often require at least 3 years of treatment. For example, Xu's study reported that 3 years of SLIT was more efficacious than 1 year or 2 years of SLIT in AR patients allergic to house dust mites (HDM).^13^ Moreover, another study suggested that 4 years of SLIT is the optimal choice for long-lasting effects in HDM-induced respiratory disease.^14^ Consistently, our data found that children obtained more beneficial results in the alleviation of clinical symptoms and medication usage at the end of 2 or 3 years of SLIT than 1 year of SLIT. However, there were no significant differences between the 2 years and 3 years groups at the end of treatment in the improvement of clinical symptoms and medication usage. This suggests that 2 and 3 years of SLIT may produce similar clinical benefits during the treatment. Next, we compared the TNSS, RMS, and SMS score 2 years at the end of the 2 years following cessation of SLIT and found that only children in the 3 years group obtained long-lasting effect, while more than 50% of the children in the 1 year and 2 years groups rebound after the cessation of treatment. These results confirm the significance of long-term SLIT in maintaining long-lasting effects.

Immune cells and inflammatory cytokines composed of complex networks are involved in the pathogenesis of AR and SLIT. Th2 cells produce IL-4 and IL-13, which contribute to the differentiation of Th2 cells and IgE production.^15^^,^^16^ In the late-phase, Th2 cytokines and tissue eosinophilia exacerbate allergic inflammation.^16^ In SLIT, IL-5^+^, IL-13^+^, and CD4^+^ cells may be used as biomarkers for successful SLIT as described in a previous study.^17^ Group 2 innate lymphoid cells (ILC2s), another main source of IL-5 and IL-13, also stimulated allergic responses and play a key role in the development of AR. Interestingly, only IL-10^+^ and ILC2s were found to influence the mechanism of allergen immunotherapy.^18^ Tfh cells also have the ability to assist B cells to produce IgE.^19^ The percentage of peripheral Tfh2 cells was positively related to IgE levels in AR.^19^ After AIT, decreased *Der* p 1-specific IL-4 Tfh cells was correlated with the alleviation of clinical symptoms.^19^ The change of specific IgE and IgG4 levels during SLIT has also received much attention, especially the serum specific IgE/total IgE ratio, which was believed to be a good biomarker for the clinical efficacy of AIT.^20^ Interestingly, our data found that the percentages of Th2, Th17, Tfh2, ILC2, and related cytokines as well as Ig levels were not changed significantly during SLIT. These discrepancies between children and adults may be explained by several possible reasons: immaturity of immune system in children leads to different immune tolerance mechanisms, different major allergen type in different study population (eg, mite vs cedar pollinosis), or the mode of AIT (SCIT vs SLIT).

In the 1 year and 2 years group, we found no change of the anti-inflammatory cytokine levels and the percentage of suppressive cells, suggesting that 1 or 2 years of SLIT may be not sufficient to induce immune tolerance. Interestingly, some previous reported immunal changes after 1 year of AIT.^21^, ^22^, ^23^, ^24^, ^25^ However, the different allergen type (mite *vs* cedar pollinosis), treatment method (SCIT *vs* SLIT), and treatment population (adult *vs* children) may explain the different results. Our data also showed that the percentages of Treg, Breg, and Tfr in the 3 years group increased significantly. Consistently, Terada et al found that the percentages of Foxp3^+^, Treg cells, and Breg cells in PBMCs were significantly elevated after 4 years of SLIT treatment for cedar pollinosis.^23^ Moreover, IL-35 induced Treg cell counts were increased in patients receiving SLIT compared to patients with AR.^26^ Tfr, a new effector subset of Treg, can limit B cell activation and IgE production.^27^ After AIT, the numbers and function of Tfr were enhanced, which is consistent with our results.^28^ Consistently, we also found that anti-inflammatory cytokines and cells were up-regulated in good responsive group compared with poor responsive groups at the end of the 2 years following cessation of SLIT. In the allergen-induced immunological responses, we also found that SLIT improved the ability of anti-inflammatory cytokines secretion and the proliferation of suppressive cells. After 3 years of SLIT, the Der p 1 induced serum IL-10, TGF-beta, IL-35 expression, and peripheral blood of Treg, Breg, Tfr ratio were increased significantly. These anti-inflammatory cytokines and suppressive cells contribute to the inhibition of allergic inflammation and corresponding symptoms.

Collectively, our data suggest that effective SLIT may be attributed to the induction of suppressive cells rather than the inhibition of inflammatory cells. Moreover, these immunological changes were not altered even two years after the cessation of the treatment, suggesting that 3 years of SLIT can establish long-term stable immune tolerance. To better describe the change of the symptoms and the immunological cells, we also examined the immunological changes every year (Data not shown). However, we chose only 3 time points to present our results for clarity because the treatment time for some group is not a full year.

## Conclusion

In summary, our results suggest that at least 3 years of SLIT treatment is necessary for long lasting effects and continued immunological improvements. During SLIT, suppressive cells such as Treg, Breg, and Tfr may play more important roles than inflammatory cells. Further studies are required to explore these results.

## Abbreviations

AR, allergic rhinitis; AIT, allergen-specific immunotherapy; SCIT, subcutaneous immunotherapy; SLIT, sublingual immunotherapy; SMS, combined symptom and medication score; TNSS, total nasal symptom score; SPT, skin-prick test; IgE, immunoglobulin E; ELISA, enzyme linked immunosorbent assay; PBMCs, peripheral blood mononuclear cells; Breg, regulatory B cell; Tfr, follicular regulatory T cell; Th2, T helper 2 cell; Tfh2, follicular T helper 2 cell; ILC2,group 2 innate lymphoid cells.

## Funding

This study was supported by grants from the National Natural Science Grant of China (No.81970861, No.82271142), the Guangdong Province Natural Science Grant (No. 2021A1515010940), the Science and Technology Program of Guangzhou (No. 202102020079, No. 202201020600, No.202201011844), Guangdong Administration of Traditional Chinese Medicine (No. 20232131) and Key Clinical Speciality of Guangzhou Women and Children's Medical Center, Guangzhou Science and technology planning project (202102010325), Guangzhou Health Science and technology project (20201A010015).

## Availability of data and materials

All data generated or analyzed during this study are included in this published article and its supplementary information files.

## Authors’ contributions

YZ and WL contributed in conception and design of the study. YZ, HX and SG contributed in acquisition of data and in analysis and interpretation of data and in drafting the article. HX, JL and CY performed the experimental work with help from QZ and XL. WL, QZ, XL, RL contributed in revising the article critically. All authors have approved the manuscript.

## Ethical statement

Our study obtained approval from the ethics committee boards of Guangzhou Women and Children's Medical Center.

## Consent for publication

The authors provide their consent for the publication of the study results.

## Declaration of competing interest

The authors declare that they have no relevant conflicts of interest.
