# Supplementary material for: Efficacy and immunological changes of sublingual immunotherapy in pediatric allergic rhinitis
Source: World Allergy Organ J. 2023 Jul 23;16(7):100803. doi: 10.1016/j.waojou.2023.100803 (PMC10382672; doi:10.1016/j.waojou.2023.100803)
Supplement: Multimedia component 1 [file mmc1.docx]

**Table S1** **Reasons for not being able to persist in SLIT treatment**

**in 1 year and 2 years group**

|  | **1 year group** | **2 years group** |
| --- | --- | --- |
| **Not satisfied with the**  **therapeutic effect** | 20 | 15 |
| **Unable to persist in treatment** | 67 | 45 |
| **Believing that further**  **treatment is not necessary** | 12 | 7 |
| **Worried about adverse events** | 2 | 3 |
| **Total** | 101 | 70 |

SLIT, sublingual immunotherapy
